# Algorithm to determine the outcome of patients with acute liver failure: a data-mining analysis using decision trees

**DOI:** 10.1007/s00535-012-0529-8

**Published:** 2012-03-09

**Authors:** Nobuaki Nakayama, Makoto Oketani, Yoshihiro Kawamura, Mie Inao, Sumiko Nagoshi, Kenji Fujiwara, Hirohito Tsubouchi, Satoshi Mochida

**Affiliations:** 1 Department of Gastroenterology and Hepatology, Faculty of Medicine, Saitama Medical University, 38 Morohongo, Moroyama-Machi, Iruma-gun, Saitama, 350-0495 Japan; 2 Department of Digestive and Life-style Related Disease, Kagoshima University Graduate School of Medical and Dental Sciences, Kagoshima, Japan; 3 Life Sciences Solutions, IBM Japan, Tokyo, Japan; 4 Yokohama Rosai Hospital for Labor Welfare Corporation, Yokohama, Japan

**Keywords:** Hepatic encephalopathy, Liver transplantation, Fulminant hepatitis, Late-onset hepatic failure

## Abstract

**Background:**

We established algorithms to predict the prognosis of acute liver failure (ALF) patients through a data-mining analysis, in order to improve the indication criteria for liver transplantation.

**Methods:**

The subjects were 1,022 ALF patients seen between 1998 and 2007 and enrolled in a nationwide survey. Patients older than 65 years, and those who had undergone liver transplantation and received blood products before the onset of hepatic encephalopathy were excluded. Two data sets were used: patients seen between 1998 and 2003 (*n*=698), whose data were used for the formation of the algorithm, and those seen between 2004 and 2007 (*n*=324), whose data were used for the validation of the algorithm. Data on a total of 73 items, at the onset of encephalopathy and 5 days later, were collected from 371 of the 698 patients seen between 1998 and 2003, and their outcome was analyzed to establish decision trees. The obtained algorithm was validated using the data of 160 of the 324 patients seen between 2004 and 2007.

**Results:**

The outcome of the patients at the onset of encephalopathy was predicted through 5 items, and the patients were classified into 6 categories with mortality rates between 23% and89%. When the prognosis of the patients in the categories with mortality rates greater than 50% was predicted as “death”, the accuracy, sensitivity, specificity, positive predictive value (PPV), and negative predictive value (NPV) of the algorithm were 79, 78, 81, 83, and 75%, respectively. Similar high values were obtained when the algorithm was employed in the patients for validation. The outcome of the patients 5 days after the onset of encephalopathy was predicted through 7 items, and a similar high accuracy was found for both sets of patients.

**Conclusions:**

Novel algorithms for predicting the outcome of ALF patients may be useful to determine the indication for liver transplantation.

## Introduction

Acute liver failure (ALF) is a clinical syndrome characterized by hepatic encephalopathy and a bleeding tendency due to the severe impairment of liver function caused by massive or submassive liver necrosis. In Japan, patients showing 40% or less of the standardized prothrombin time value or INRs of 1.5 or more caused by severe liver damage within 8 weeks of onset of the symptoms are diagnosed as having ALF, where the liver function prior to the current onset of liver damage was estimated to be normal [1]. ALF is classified into the categories of “acute liver failure without hepatic coma” and “acute liver failure with hepatic coma,” depending on the severity of the hepatic encephalopathy; the latter is further classified into 2 types, the “acute type” and the “subacute type”, in which grade II or more severe hepatic coma develops within 10 days and between 11 and 56 days, respectively, after the onset of disease symptoms. Also, patients with less than 40% of the standardized prothrombin time value or INRs of 1.5 or more and grade II or more severe hepatic coma occurring between 8 and 24 weeks of the onset of disease symptoms are diagnosed as having late-onset hepatic failure (LOHF), as a disease related to ALF. ALF in Japan has been typically regarded as fulminant hepatitis, for which the diagnostic criteria were established by the Inuyama Symposium held in 1981 [2]. Among patients with ALF, those showing histological findings of hepatitis (characterized by inflammatory lymphocyte infiltration), as well as 40% or less of the standardized prothrombin timeand grade II or more severe hepatic encephalopathy, are diagnosed as having “fulminant hepatitis”, which is classified as acute and subacute types in the same manner as ALF [2, 3]. Thus, fulminant hepatitis is almost synonymous with ALF in the United States and Europe as well as in Japan, except that patients without histological evidence of hepatitis are excluded from both disease conditions in Japan. Thus, ALF caused by viral infections, autoimmune hepatitis, and drug allergy-induced liver injury is included in the diagnosis of fulminant hepatitis, while ALF caused by drug/chemical intoxication (such as acetaminophen intoxication) microcirculatory disturbances, Wilson’s disease, acute fatty liver of pregnancy, and Reye’s syndrome is excluded from that. A history of chronic liver disease preceding the onset of acute liver injury also precludes the diagnosis of fulminant hepatitis and LOHF, while inactive hepatitis B virus (HBV) carriers showing normal serum alanine aminotransferase (ALT) values before acute exacerbation of hepatitis are included in both these disease conditions.

According to a nationwide survey conducted by the Intractable Liver Diseases Study Group of Japan constituted under the aegis of the Ministry of Health, Welfare and Labour [4], artificial liver support with plasma exchange and/or hemodiafiltration was performed in almost all patients with fulminant hepatitis and LOHF between 1998 and 2003. Also, about 70 and 60% of the patients, respectively, received intravenous glucocorticoid treatment and anticoagulant therapy with an antithrombin III concentrate. Moreover, patients with HBV infection have received antiviral therapy with lamivudine or entecavir since 1998. Despite the use of these therapeutic modalities, however, the outcome of the patients receiving these treatments had not improved; the survival rates of patients with the acute and subacute types of fulminant hepatitis not treated with liver transplantation were 54 and 24%, respectively, , and in those with LOHF not treated with liver transplantation the survival rate was 12% [4]. In contrast, the outcome of the patients receiving liver transplantation was excellent, with the survival rate being 78% among those with fulminant hepatitis and 75% among those with LOHF, suggesting that liver transplantation is the optimal therapeutic strategy for the rescue of patients with ALF, irrespective of the disease types in Japan.

The indications for liver transplantation in patients with ALF are currently determined according to the guideline published by the Acute Liver Failure Study Group of Japan in 1996 [5, 6]. The predictive accuracy, however, decreased when the guideline was adopted for patients seen between 1998 and 2003; the accuracy values in the patients not receiving liver transplantation were 67 and 78% among those with the acute and subacute types of fulminant hepatitis, respectively, and the specificity of the guideline was extremely low especially in patients with the subacute type of fulminant hepatitis [6]. Thus, the guideline to determine the indication for liver transplantation in ALF patients in Japan needs to be updated.

Recently, we performed a cluster analysis of the patients with fulminant hepatitis and LOHF seen between 1998 and 2007 to evaluate the validity of the classification of ALF in Japan [7]. We adopted the self-organizing map (SOM), one of the data-mining methods introduced by Kohonen as an artificial neural network [8], which has been shown to be suitable for analyses of complex multidimensional relationships in various medical science fields [9–15]. Consequently, we found that ALF patients could be classified into three clusters independent of the interval between the onset of disease symptoms and the development of hepatic encephalopathy, and the outcome of the patients differed markedly among the clusters [7]. These observations prompted us to postulate that data-mining methods may be useful to revise the above-mentioned guideline.

We report on algorithms to predict the outcome of ALF patients under intensive medical care without liver transplantation; these algorithms were established based on the data-mining analysis using decision trees. The algorithms were constructed using the data from ALF patients without liver transplantation, because there may have been many patients among those receiving liver transplantation who could have been rescued by intensive medical care.

## Patients and methods

### Patients

The subjects of this study were 1,022 patients with ALF who were enrolled in the nationwide survey of fulminant hepatitis and LOHF conducted by the Intractable Hepato-Biliary Disease Study Group of Japan between 1999 and 2008 (formerly the Intractable Liver Diseases Study Group of Japan, before 2003). All of the patients showed grade II or more severe hepatic encephalopathy and prothrombin times of less than 40% of the standardized value and were admitted to 610 hospitals specializing in hepatology in Japan between 1998 and 2007. Patients without histological evidence of hepatitis, such as those with hepatitis due to drug-toxicity, circulatory disturbance, and metabolic diseases, were excluded from the analysis. The interval between the onset of the hepatitis symptoms and the development of encephalopathy was 10 days or less in 472 patients (group-A; acute type of fulminant hepatitis), between 11 and 56 days in 468 patients (group-B; subacute type of fulminant hepatitis), and more than 56 days in 82 patients (group-C; LOHF). The patients were classified into two data sets; 698 patients (316, 318, and 64 in group-A, group-B, and group-C, respectively) seen between 1998 and 2003, and 324 patients (156, 150, and 18, respectively, in each group) seen between 2004 and 2007. The former data set was used for the formation of the algorithms to predict the outcome of the patients and the latter data set was used for the validation of the established algorithms. The clinical features of all patients were obtained until either of the following time-points: they died in hospital, or received liver transplantation, or were discharged following improvement of liver function; the outcomes of the patients were expressed as “dead”, “transplanted”, and “rescued”, respectively. Missing data were managed through available-case analysis, in which all relevant data were used.

The etiology of ALF was determined based on the definition proposed by the Intractable Liver Diseases Study Group of Japan constituted under the aegis of the Ministry of Health, Welfare and Labour [1, 4]. Criteria for complications were defined as follows: *Infection*; (1) manifestation of organic symptoms and/or imaging findings, (2) body temperature of 38°C or more, (3) white blood cell counts of 10,000 cells/mm^3^ or more, (4) positive for causative bacteria in organs suspicious of infection and/or increase of white blood cell counts in body fluid. Patients were diagnosed as having infection when two or more of these criteria were present. *Brain edema*; (1) typical findings on computed tomography (CT) images, or (2) intracranial pressure of 25 mmHg or more. *Gastrointestinal bleeding*; (1) hematemesis and/or drainage of blood from a catheter in the upper gastrointestinal tract, (2) tarry stool or melena, (3) endoscopic findings of bleeding. Patients were diagnosed as having gastrointestinal bleeding when one or more of these criteria were present. *Renal failure*; (1) urine volume output of 400 mL or less per day, or (2) serum creatinine levels of 2.0 mg/dL or higher. *Disseminated intravascular coagulation* (*DIC*); patients were diagnosed as having DIC when the score on the scoring system for DIC revised by the Japanese Association for Acute Medicine (JAAM) [16] was four or more. *Heart failure*; (1) chest X-ray showing an enlarged cardiac silhouette, (2) chest X-ray showing pulmonary congestion, (3) an ejection fraction of 40% or less. Patients were diagnosed as having heart failure when two or more of these criteria were present. Atrophy of the liver was assessed by each practitioner subjectively based on imaging through ultrasound and/or CT scan examinations.

The demographic and clinical features, the therapies undertaken, and the consequent outcomes of the patients are shown in the various sections of Table 1. Of the total study population seen between 1998 and 2007, 40.2% had underlying diseases such as metabolic syndrome, and most of such patients were on daily medications. The etiology of hepatitis was viral infection in 69.3, 31.2, and 17.1% of the patients in group-A, group-B, and group-C, respectively. In most cases, the causative virus was hepatitis B virus (HBV); transient infection was predominant in the patients in group-A, whereas inactive carriers showing acute exacerbation of hepatitis predominated in group-B. The etiology was indeterminate in 41.5 and 47.6% of the patients in group-B and group-C, respectively. Autoimmune hepatitis and drug-induced liver injury were found in 12.0 and 13.0%, respectively, of the patients in group-B, and in 17.1 and 15.9%, respectively, of those in group-C. The survival rates of the 811 patients who were treated conservatively without liver transplantation were 53.4, 24.5, and 12.1%, respectively, in group-A, group-B, and group-C patients. The remaining 211 patients (20.6%) underwent liver transplantation, and the survival rates were 56.4, 39.7, and 25.6%, respectively, in the patients in group-A, group-B, and group-C.

**Table 1:** Demographic and clinical features of acute liver failure patients in Japan seen between 1998 and 2003 and those seen between 2004 and 2007

(a) Demographic features and the etiology of acute liver failure				
1998–2003	Total (*n* = 698)	Group-A^a^ (*n* = 316)	Group-B (*n* = 318)	Group-C (*n* = 64)
Male:female (:unknown)^b^	346:351 (:1)	167:148 (:1)	152:166	27:37
Age (years)^c^	47.0 ± 16.8^†^	45.1 ± 16.6^†^	47.8 ± 17.1^†^	51.9 ± 15.0^†^
HBV carrier^d^	14.1 (93/658)*	12.7 (37/291)*	17.4 (53/305)	4.8 (3/62)
Underlying diseases^d, e^	38.5 (265/689)	32.7 (102/312)	41.5 (130/313)	51.6 (33/64)
History of medication^d^	42.0 (282/672)*	36.6 (112/306)*	45.7 (139/304)*	50.0 (31/62)
Etiology^d^				
Viral infection	48.0 (335)	71.2 (225)	31.8 (101)	14.1 (9)
HAV	6.4 (45)^#^	12.0 (38)	1.9 (6)	1.6 (1)
HBV	38.8 (271)	56.6 (179)	27.0 (86)	9.4 (6)
Transient infection	23.2 (162)	41.8 (132)	8.8 (28)	3.1 (2)
Carrier	13.5 (94)	12.0 (38)	16.7 (53)	4.7 (3)
Undetermined	2.1 (15)^#^	2.8 (9)^#^	1.6 (5)	1.6 (1)
HCV	1.4 (10)	1.6 (5)	1.3 (4)	1.6 (1)
HEV	0.4 (3)	0 (0)^#^	0.9 (3)	0 (0)
Other virus	0.9 (6)	0.9 (3)	0.6 (2)	1.6 (1)
Autoimmune hepatitis	6.9 (48)	1.6 (5)	10.7 (34)	14.1 (9)
Drug allergy-induced	9.3 (65)^#^	6.0 (19)^#^	11.3 (36)	15.6 (10)
Indeterminate	32.8 (229)	18.7 (59)	42.8 (136)	53.1 (34)
Insufficient examinations^f^	3.0 (21)^#^	2.5 (8)	3.5 (11)	3.1 (2)

2004–2007	Total (*n* = 324)	Group-A^a^ (*n* = 156)	Group-B (*n* = 150)	Group-C (*n* = 18)
Male:female ^b^	152:172	82:74	64:86	6:12
Age (years)^c^	51.1 ± 16.1	48.6 ± 15.5	52.7 ± 16.5	60.3 ± 11.5
HBV carrier^d^	11.7 (33/282)	9.5 (12/126)	13.7 (19/139)	11.8 (2/17)
Underlying diseases^d, e^	44.0 (139/316)	39.7 (60/151)	47.6 (70/147)	50.0 (9/18)
History of medication^d^	60.3 (184/305)	51.7 (75/145)	66.9 (95/142)	77.8 (14/18)
Etiology^d^				
Viral infection	46.9 (152)	65.4 (102)	30.0 (45)	33.3 (6)
HAV	3.1 (10)	6.4 (10)	0.0 (0)	0.0 (0)
HBV	41.0 (133)	56.4 (88)	26.7 (40)	27.8 (5)
Transient infection	21.9 (71)	38.5 (60)	6.7 (10)	5.6 (1)
Carrier	12.3 (40)	6.4 (10)	18.0 (27)	16.7 (3)
Undetermined	6.8 (22)	11.5 (18)	2.0 (3)	5.6 (1)
HCV	0.9 (3)	0.6 (1)	1.3 (2)	0.0 (0)
HEV	1.2 (4)	1.3 (2)	1.3 (2)	0.0 (0)
Other virus	0.6 (2)	0.6 (1)	0.7 (1)	0.0 (0)
Autoimmune hepatitis	9.9 (32)	3.2 (5)	14.7 (22)	27.8 (5)
Drug allergy-induced	14.5 (47)	12.2 (19)	16.7 (25)	16.7 (3)
Indeterminate	27.8 (90)	17.3 (27)	38.7 (58)	27.8 (5)
Insufficient examinations^f^	0.9 (3)	1.9 (3)	0.0 (0)	0.0 (0)

(b) Complications of acute liver failure^g^				
1998–2003	Total (*n* = 698)	Group-A^a^ (*n* = 316)	Group-B (*n* = 318)	Group-C (*n* = 64)
Infection	39.1 (247/632)	35.0 (100/286)	40.8 (117/287)	50.8 (30/59)
Brain edema	31.0 (173/558)*	35.3 (91/258)*	29.0 (73/252)*	18.8 (9/48)
Gastrointestinal bleeding	20.1 (134/668)	22.2 (67/302)*	16.7 (51/305)	26.2 (16/61)
Renal failure	36.5 (249/682)	41.5 (129/311)*	29.9 (92/308)	44.4 (28/63)
DIC	41.5 (271/653)	43.4 (129/297)*	41.3 (124/300)	33.9 (19/56)
Congestive heart failure	10.5 (70/664)*	11.2 (34/303)	9.6 (29/301)*	11.7 (7/60)

2004–2007	Total (*n* = 324)	Group-A^a^ (*n* = 156)	Group-B (*n* = 150)	Group-C (*n* = 18)
Infection	35.7 (109/305)	33.8 (49/145)	35.9 (51/142)	50.0 (9/18)
Brain edema	16.7 (47/282)	20.1 (28/139)	11.7 (15/128)	26.7 (4/15)
Gastrointestinal bleeding	15.4 (48/312)	12.5 (19/152)	17.4 (25/144)	25.0 (4/16)
Renal failure	35.4 (113/319)	35.7 (55/154)	35.4 (52/147)	33.3 (6/18)
DIC	35.1 (108/308)	30.6 (45/147)	37.1 (53/143)	55.6 (10/18)
Congestive heart failure	7.6 (23/303)	8.7 (13/150)	5.8 (8/137)	12.5 (2/16)

(c) Therapeutic strategies undertaken following the onset of hepatic encephalopathy^g^				
1998–2003	Total (*n* = 698)	Group-A^a^ (*n* = 316)	Group-B (*n* = 318)	Group-C (*n* = 64)
Glucocorticoids	67.6 (470/695)	60.5 (190/314)	76.0 (241/317)	75.0 (48/64)
Glucagon/insulin	43.2 (300/694)*	37.6 (118/314)*	47.5 (150/316)*	50.0 (32/64)*
BCAA-rich solution	32.9 (227/689)*	27.6 (86/312)	35.8 (112/313)*	45.3 (29/64)
Plasma exchange	91.1 (634/696)	90.1 (283/314)	93.4 (297/318)	84.4 (54/64)
Hemodiafiltration	74.7 (518/693)	75.2 (236/314)	77.2 (244/316)	60.3 (38/63)
Prostaglandin E1	23.2 (160/691)*	19.4 (61/314)*	25.8 (81/314)*	28.6 (18/63)*
Cyclosporin A	13.9 (96/691)*	11.1 (35/314)	15.9 (50/314)	17.5 (11/63)
Interferon	19.5 (135/691)*	22.0 (69/314)*	19.7 (62/314)*	6.3 (4/63)
Nucleoside analog	23.9 (164/687)*	30.9 (96/311)*	20.4 (64/314)*	6.5 (4/62)*
Anticoagulation therapy	59.6 (413/693)*	57.3 (180/314)*	60.1 (190/316)	68.3 (43/63)*
Liver transplantation	20.3 (142/698)	14.6 (46/316)	26.4 (84/318)*	18.8 (12/64)

2004–2007	Total (*n* = 324)	Group-A^a^ (*n* = 156)	Group-B (*n* = 150)	Group-C (*n* = 18)
Glucocorticoids	71.8 (232/323)	66.7 (104/156)	75.8 (113/149)	83.3 (15/18)
Glucagon/insulin	15.5 (50/323)	16.7 (26/156)	14.1 (21/149)	16.7 (3/18)
BCAA-rich solution	23.7 (76/321)	18.2 (28/154)	26.2 (39/149)	50.0 (9/18)
Plasma exchange	90.7 (293/323)	92.3 (144/156)	91.3 (136/149)	72.2 (13/18)
Hemodiafiltration	69.9 (225/322)	69.7 (108/155)	73.8 (110/149)	38.9 (7/18)
Prostaglandin E1	7.4 (24/323)	7.7 (12/156)	7.4 (11/149)	5.6 (1/18)
Cyclosporin A	9.0 (29/323)	6.4 (10/156)	11.4 (17/149)	11.1 (2/18)
Interferon	13.3 (43/323)	14.7 (23/156)	12.1 (18/149)	11.1 (2/18)
Nucleoside analog	39.1 (126/322)	51.6 (80/155)	27.5 (41/149)	27.8 (5/18)
Anticoagulation therapy	45.5 (147/323)	39.1 (61/156)	54.4 (81/149)	27.8 (5/18)
Liver transplantation	21.3 (69/324)	12.8 (20/156)	30.0 (45/150)	22.2 (4/18)

(d) The outcome of the patients^g^				
1998–2003	Total (*n* = 698)	Group-A^a^ (*n* = 316)	Group-B (*n* = 318)	Group-C (*n* = 64)
Survival rate	45.6 (318/698)	56.3 (178/316)	39.3 (125/318)	23.4 (15/64)
Treated without liver transplantation	37.4 (208/556)	53.7 (145/270)	24.4 (57/234)	11.5 (6/52)
Treated with liver transplantation	77.5 (110/142)	71.7 (33/46)	81.0 (68/84)	75.0 (9/12)

2004–2007	Total (*n* = 324)	Group-A^a^ (*n* = 156)	Group-B (*n* = 150)	Group-C (*n* = 18)
Survival rate	47.8 (155/324)	56.4 (88/156)	40.7 (61/150)	33.3 (6/18)
Treated without liver transplantation	39.2 (100/255)	52.9 (72/136)	24.8 (26/105)	14.3 (2/14)
Treated with liver Transplantation	79.7 (55/69)	80.0 (16/20)	77.8 (35/45)	100.0 (4/4)

*HBV* hepatitis B virus, *HAV* hepatitis A virus, *HCV* hepatitis C virus, *HEV* hepatitis E virus, *BCAA* branched-chain amino acid, *DIC* disseminated intravascular coagulation

^a^The interval between the onset of the hepatitis symptoms and the onset of grade II or more severe hepatic encephalopathy was 10 days or less (group-A), between 11 and 56 days (group-B), and more than 56 days (group-C)

^b^Number of patients

^c^Mean ± SD

^d^The values are the percentages of patients (%), and the values in parentheses represent the numbers of patients for the calculation of the percentage

^e^Diseases such as metabolic syndrome, malignancy, and psychiatric disorders

^f^The etiology was unknown because of insufficient examinations

^g^The values are the percentages of patients (%), and the values in parentheses represent the numbers of patients for calculation of the percentage

^†^ *p* < 0.05 versus 2004–2007 by Student’s *t*-test

*^^ *p* < 0.05 versus 2004–2007 by the χ^2^ test

^#^ *p* < 0.05 versus 2004–2007 by the χ^2^ test and analysis of residuals in cross tabulation

The demographic and clinical features in the patients seen between 1998 and 2003 and those seen between 2004 and 2007 were similar, except for the following items (Table 1a): the ages of the patients seen between 2004 and 2007 were significantly higher than the ages in those seen between 1998 and 2003 irrespective of the groups to which they belonged . On the other hand, the percentage of HBV carriers in group-A was greater in patients seen between 1998 and 2003 compared to the percentage in those seen between 2004 and 2007. In contrast, the percentages of patients with previous medication in group-A and group-B were greater in those seen between 2004 and 2007 than in those seen between 1998 and 2003. There were also differences in the incidence of brain edema and congestive heart failure between patients seen between 1998 and 2003 and those seen between 2004 and 2007 (Table 1b). Also, the percentages of patients who received therapies such as glucagon and insulin infusion, administration of branched-chain-rich amino acid, prostaglandin E1, interferon, and nucleoside analogs for HBV, and anticoagulant therapies, were different between the two data sets (Table 1c). However, the survival rates of patients both with and without liver transplantation were equivalent in the two data sets (Table 1d).

The following patients were excluded from both data sets: (1) patients older than 65 years; (2) those who had undergone liver transplantation; and (3) those who had received blood product administration before the onset of hepatic encephalopathy. Patients aged more than 65 years were excluded from the analysis because the Act on Organ Transplantation (Law number: Act No. 104 of 1997) recommends that liver transplantation recipients should be younger than 60 years, and in general, in Japan, liver transplantation has been done in recipients aged 65 years or less. Consequently, the data of 371 patients (male 196, female 175) aged between 2 and 65 years (mean ± SD 44.1 ± 14.2) seen between 1998 and 2003 were used for the formation of the algorithms. The disease types of these patients were group-A, group-B, and group-C in 206, 140, and 25 patients, respectively. Validation of the established algorithms was performed in 160 patients (male 81, female 79), aged between 17 and 65 years (47.5 ± 11.9), seen between 2004 and 2007 (98, 56, and 6 patients in group-A, group-B, and group-C, respectively). The algorithms were also employed for the 211 patients who had received liver transplantation between 1998 and 2007, comprising 80 male and 131 female patients aged between 7 and 70 years (39.6 ± 15.6), with 66, 129, and 16 patients belonging to group-A, group-B, and group-C, respectively.

### Formation of the algorithms through decision tree analysis

Two types of algorithms were formed using the different data sets; one for the prediction of the patients’ outcome at the onset of hepatic encephalopathy of grade II or more (day 0), and the other for the prediction 5 days later (day 5). Data on a total of 62 items, including: (1) the demographic features of the patients, (2) clinical features and laboratory and imaging data at the onset of hepatic encephalopathy, and (3) the therapies received until the development of hepatic encephalopathy, were collected from 371 patients seen between 1998 and 2003 (Table 2), and used for the formation of the algorithm predicting the patients’ outcome on day 0. Data on a total of 73 items, including 62 items for the algorithm predicting the patients’ outcome on day 0, and clinical features, laboratory and imaging data, and the therapies received at 5 days after the onset of hepatic encephalopathy, collected from the same patients, were used for the formation of the algorithm predicting the patients’ outcome on day 5. Items such as age, body weight, and biochemical data were analyzed as continuous variables, while those such as gender, outcomes, and complications were analyzed as nominal variables.

**Table 2:** Items characteristic of acute liver failure patients used in the decision tree analysis to establish the algorithms

(a) Items for construction of the algorithm for the patients at the onset of hepatic encephalopathy (day 0)
The types of hepatitis: acute and subacute types of fulminant hepatitis and LOHF
Outcomes: survived and died among patients treated conservatively without liver transplantation and the patients who underwent transplantation
Gender: male and female
Age (years, continuous variable)
Complications preceding acute liver failure: diseases different from liver diseases such as metabolic syndrome, psychiatric diseases, and malignancies
HBV carrier
Past medical history: operations, blood infusions, alcohol intake, and medications
Family history: liver diseases
Etiology of hepatitis: viral infection [HAV, HBV (transient infection, carrier, undetermined), HCV, HEV, other virus], autoimmune hepatitis, drug-induced, indeterminate, and unknown due to insufficient examinations
Interval between the onset of the hepatitis symptoms and the subsequent events (days, continuous variables): onset of jaundice and grade II or more severe hepatic encephalopathy
Interval between the onset of jaundice and the subsequent events (days, continuous variables): onset of hepatic encephalopathy of grade II or more
Symptoms at the onset of grade II or more severe hepatic encephalopathy: fever, jaundice, ascites, edema, flapping tremor, halitosis, loss of liver dullness, convulsion, tachycardia, and hyperventilation
Laboratory data at the onset of grade II or more severe hepatic encephalopathy (continuous variables): the grading of the encephalopathy, peripheral counts of WBC and platelets, prothrombin time, hepaplastin test, plasma concentrations of antithrombin III and ammonia, serum concentrations of AST, ALT, total albumin, bilirubin, AFP, and HGF, the serum concentration ratios of direct to total bilirubin, molar ratio of BCAA to tyrosine (BTR), and Fischer ratio
Atrophy of the liver at the onset of grade II or more severe hepatic encephalopathy
Complications of acute liver failure at the onset of grade II or more severe hepatic encephalopathy: bacterial and fungal infections, gastrointestinal bleeding, renal failure, cardiac failure, disseminated intravascular coagulation, other complications
Number of complications at the onset of grade II or more severe hepatic encephalopathy (continuous variables)
The therapies received: plasma exchange, hemodiafiltration, glucocorticoids, glucagon and insulin, prostaglandin E1, interferon, lamivudine or entecavir, cyclosporin A, anticoagulants, and fresh-frozen plasma
(b) Items for construction of the algorithm for the patients at 5 days after the onset of hepatic encephalopathy (day 5)
The types of hepatitis: acute and subacute types of fulminant hepatitis and LOHF
Outcomes: survived and died among patients treated conservatively without liver transplantation and the patients who underwent transplantation
Gender: male and female
Age (years, continuous variable)
Complications preceding acute liver failure: diseases different from liver diseases such as metabolic syndrome, psychiatric diseases, and malignancies
HBV carrier
Past medical history: operations, blood infusions, alcohol intake, and medications
Family history: liver diseases
Etiology of hepatitis: viral infection [HAV, HBV (transient infection, carrier, undetermined), HCV, HEV, other virus], autoimmune hepatitis, drug-induced, indeterminate, and unknown due to insufficient examinations
Interval between the onset of the hepatitis symptoms and the subsequent events (days, continuous variables): onset of jaundice and grade II or more severe hepatic encephalopathy
Interval between the onset of jaundice and the subsequent events (days, continuous variables): onset of hepatic encephalopathy of grade II or more
Symptoms at the onset of grade II or more severe hepatic encephalopathy: fever, jaundice, ascites, edema, flapping tremor, halitosis, loss of liver dullness, convulsion, tachycardia, and hyperventilation
Laboratory data at the onset of grade II or more severe hepatic encephalopathy (continuous variables): the grading of the encephalopathy, peripheral counts of WBC and platelets, prothrombin time, hepaplastin test, plasma concentrations of antithrombin III and ammonia, serum concentrations of AST, ALT, total albumin, bilirubin, AFP, and HGF, the serum concentration ratios of direct to total bilirubin, molar ratio of BCAA to tyrosine (BTR), and Fischer ratio
Symptoms and laboratory data 5 days after the onset of encephalopathy (continuous variables): the grading of the encephalopathy, prothrombin time
Atrophy of the liver at the onset of grade II or more severe hepatic encephalopathy and 5 days later Complications of acute liver failure at the onset of grade II or more severe hepatic encephalopathy: Bacterial and fungal infections, gastrointestinal bleeding, renal failure, cardiac failure, disseminated intravascular coagulation, other complications
Number of complications at the onset of grade II or more severe hepatic encephalopathy and 5 days later (continuous variables) Complications of acute liver failure 5 days after the onset of encephalopathy: bacterial and fungal infections, gastrointestinal bleeding, renal failure, cardiac failure, disseminated intravascular coagulation, other complications
Number of complications 5 days after the onset of encephalopathy (continuous variables)
The therapies received: plasma exchange, hemodiafiltration, glucocorticoids, glucagon and insulin, prostaglandin E1, interferon, lamivudine or entecavir, cyclosporin A, anticoagulants, fresh-frozen plasma, and liver transplantation

*LOHF* late-onset hepatic failure, *HAV* hepatitis A virus, *HBV* hepatitis B virus, *HCV* hepatitis C virus, *HEV* hepatitis E virus *WBC* white blood cell count, *AST* aspartate aminotransferase, *ALT* alanine aminotransferase, *AFP* alpha-fetoprotein, *HGF* hepatocyte growth factor, *BCAA* branched-chain amino acids

The decision tree analysis was performed using Intelligent Miner software (IBM, Armonk, New York, USA), which can automatically search a data set to find the optimal classification variables leading to the building of a decision tree algorithm [15]. Briefly, all items derived from the patients were evaluated to determine which variables and cutoff points might produce the most significant division into two subgroups showing mortality divergent from each other. Then the same analytic procedures were applied to all newly defined subgroups. These procedures were repeated and were terminated when either no additional significant variables were detected or when the sample size decreased to less than 20.

### Evaluation of the established algorithms

The usefulness of the established algorithms was assessed through the following evaluations: (1) comparison of the mortality rates in patients belonging to each category to observe differences between patients used for the formation and those used for the validation of the algorithms; (2) the predictive accuracies, sensitivity, specificity, and positive and negative predictive values (PPV and NPV) among patients for both the formation and the validation of the algorithms, calculated based on the postulation that the outcome of the patients in the categories with mortality rates greater than 50% was predicted as “death”; and (3) the distribution of the patients in each category, when the data of the patients receiving liver transplantation were applied for the algorithms.

In each evaluation, data on the totals of 62 and 73 items, respectively, were selected for the algorithm at the onset of hepatic encephalopathy and that at 5 days after the development of encephalopathy, in a similar manner to the selection of data for the formation of the algorithms.

### Statistical analysis

Statistical testing was performed using SPSS version 15.0J (SPSS, Tokyo, Japan). Results are expressed as means ± SD. Continuous variables were compared using Student’s *t-*test. Categorical data were compared using the χ^2^ test and analysis of residuals in cross tabulation.

## Results

### Algorithms to predict the outcome of patients with ALF based on decision tree analysis

**Fig. 1:**
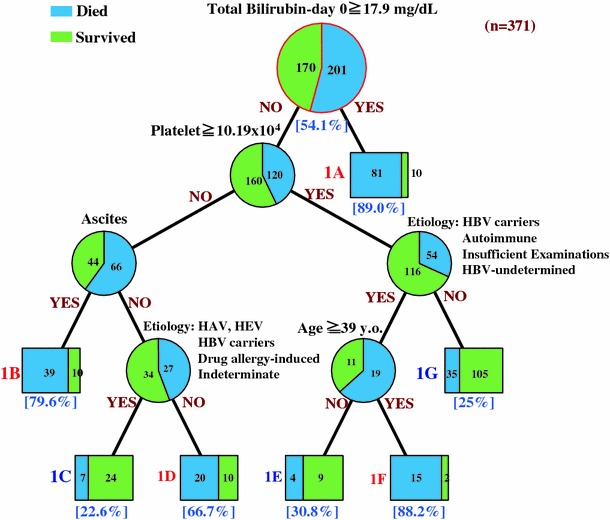
The decision tree algorithm for outcome prediction at the onset of grade II or more severe hepatic encephalopathy (day 0). *HBV* hepatitis B virus, *HAV* hepatitis A virus, *HEV* hepatitis E virus

Three hundred and seventy-one patients with ALF were classified through 5 items into 6 categories on the decision tree based on the data set obtained at the onset of hepatic encephalopathy (day 0) (Fig. 1). The mortality rate of patients with a serum bilirubin concentration of greater than 17.9 mg/dL was 89% (category-1A: *n* = 91). Two hundred and eighty patients with bilirubin concentrations of less than 17.9 mg/dL were divided into 2 groups according to peripheral blood platelet counts and further divided into 6 category groups according to age, the presence of ascites, and the disease etiology. The mortality rate of patients showing peripheral blood platelet counts of less than 10.2 × 10^4^/mm^3^ with ascites was 80% (category-1B: *n* = 49). In contrast, 61 patients with peripheral blood platelet counts of less than 10.2 × 10^4^/mm^3^ without ascites were divided into 2 groups according to the disease etiology; the mortality rate of patients with disease due to hepatitis A virus (HAV) and hepatitis E virus (HEV) infection and drug-allergy induced hepatitis, HBV carriers showing acute hepatitis exacerbation, and those with indeterminate etiology was 23% (category-1C: *n* = 31), whereas the mortality rate of those with other etiologies was 67% (category-1D: *n* = 30). The remaining 170 patients showing platelet counts of 10.2 × 10^4^/mm^3^ or more were divided into 2 groups according to the different classification criteria of disease etiology; the mortality rates of HBV carriers showing acute hepatitis exacerbation and patients with autoimmune hepatitis were 31% (category-1E: *n* = 13) if the patient age was less than 39 years and 88% (category-1F: *n* = 17) if the age was 39 years old or more, whereas the mortality rate of those with disease due to other etiologies was 25% (category-1G: *n* = 140).

**Fig. 2:**
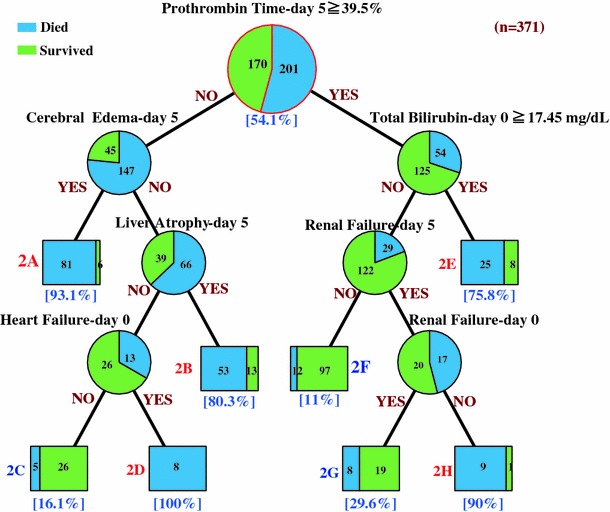
The decision tree algorithm for outcome prediction at 5 days after the onset of grade II or more severe hepatic encephalopathy (day 5)

Based on the data set obtained 5 days after the onset of hepatic encephalopathy (day 5), ALF patients were classified through 7 items into 8 categories (Fig. 2). First, the patients were divided into 2 groups according to prothrombin time at 5 days after the development of encephalopathy. One hundred and ninety-two patients showing a prothrombin time of less than 39.5% of the standardized value were further classified through the presence of brain edema, liver atrophy, and cardiac failure at 5 days after the onset of encephalopathy. The mortality rate of patients with brain edema was 93% (category-2A: *n* = 87), but those without brain edema showed mortality rates of 80% (category-2B: *n* = 66), 16% (category-2C: *n* = 31), and 100% (category-2D: *n* = 8), respectively, when liver atrophy was present, both liver atrophy and cardiac failure were absent, and cardiac failure was present despite the absence of liver atrophy. In contrast, 179 patients showing a prothrombin time of 39.5% or more of the standardized value were classified by the serum bilirubin concentration. The mortality rate of the patients showing serum bilirubin concentrations of 17.45 mg/dL or more was 76% (category-2E: *n* = 33), whereas those with a serum bilirubin concentration of less than 17.45 mg/dL were further classified based on the presence of renal failure both at the onset of hepatic encephalopathy and 5 days later. The mortality rate of the patients without renal failure at 5 days after the onset of the encephalopathy was 11% (category-2F: *n* = 109). In contrast, the mortality rates of those with renal failure at 5 days were 30% (category-2G: *n* = 27) and 90% (category-2H: *n* = 10), respectively, depending on the presence and absence of renal failure at the onset of the encephalopathy.

As shown in Table 3, the predictive accuracies assessed in patients for the establishment of the algorithms were 79% at the onset of hepatic encephalopathy and 84% at 5 days after the onset of encephalopathy, when the estimated prognosis of patients classified in categories-1A, -1B, -1D, and -1F and categories-2A, -2B, -2D, -2E, and -2H was determined as “death”. The sensitivity, specificity, PPV, and NPV were 78, 81, 83, and 75%, respectively, at the onset of the encephalopathy, and 83, 85, 87, and 81%, respectively, at 5 days later.

**Table 3:** The accuracy of the decision tree algorithms to predict the prognostic outcome of acute liver failure patients at the onset of hepatic encephalopathy and 5 days later

	At the onset of hepatic encephalopathy	At 5 days after the onset of hepatic encephalopathy
Patients for the formation of the algorithm 1998–2003 (*n* = 371)		
Accuracy	79.0	83.6
Sensitivity	77.6	82.6
Specificity	80.6	84.7
PPV	82.5	86.5
NPV	75.3	80.5
Patients for the validation of the algorithm 2004–2007 (*n* = 160)		
Accuracy	71.2	73.1
Sensitivity	75.0	63.6
Specificity	67.1	82.4
PPV	70.6	77.8
NPV	71.8	70.0

*PPV* positive predictive value, *NPV* negative predictive value

### Validation of the established algorithms

One hundred and sixty patients with ALF, seen between 2004 and 2007, were classified into 7 categories through the analysis using the data set at the onset of hepatic encephalopathy, and 8 categories using the data set at 5 days after the onset of encephalopathy. The number of patients who died and the mortality rates of the patients in each category are shown in Table 4. The distribution of the patients and the mortality rates in each category were almost equivalent to those in the patients used for the formation of the algorithms both at the onset of hepatic encephalopathy and 5 days later, except for category-2C. The mortality rate in patients classified as category-2C was 16.1% in patients used for the formation of the algorithm, while the rate was 91.7% in those used for the validation (Table 4b).

**Table 4:** The numbers of deaths and the mortality rates of patients in each category classified through decision tree analysis: comparison among patients used for the formation of the algorithm, those used for the validation of the algorithm, and those who received liver transplantation

Categories classified through decision tree analysis	Mortality rates of patients % (number of patients)		Number of patients
	Patients for algorithm formation 1998–2003 (*n* = 371)	Patients for algorithm validation 2004–2007 (*n* = 160)	Patients receiving liver transplantation 1998–2007 (*n* = 211)
(a) The algorithm for the patients at the onset of hepatic encephalopathy			
1A	89.0 (81/91)	83.9 (26/31)	95
1B	79.6 (39/49)	50.0 (16/32)	34
1C	22.6 (7/31)	37.5 (3/8)	10
1D	66.7 (20/30)	83.3 (10/12)	8
1E	30.8 (4/13)	18.2 (2/11)	7
1F	88.2 (15/17)	80.0 (8/10)	4
1G	25.0 (35/140)	30.2 (16/53)	53
(b) The algorithm for the patients at 5 days after the onset of hepatic encephalopathy			
2A	93.1 (81/87)	86.4 (19/22)	19
2B	80.3 (53/66)	71.4 (15/21)	36
2C	16.1 (5/31)	91.7 (11/12)	16
2D	100.0 (8/8)	– (0/0)	0
2E	75.8 (25/33)	72.7 (8/11)	18
2F	11.0 (12/108)	17.3 (9/52)	20
2G	29.6 (8/27)	25.0 (4/16)	1
2H	90.0 (9/10)	– (0/0)	2

The predictive accuracies assessed in patients for validation of the algorithms were 71 and 73%, respectively, at the onset of hepatic encephalopathy and 5 days later, similar to findings in the patients used for the formation of the algorithms (Table 3). The sensitivity, specificity, PPV, and NPV were 75, 67, 71, and 72%, respectively, at the onset of the encephalopathy, and 64, 82, 78, and 70%, respectively, at 5 days after the onset of encephalopathy.

### Application of the algorithms for ALF patients receiving liver transplantation

When the data from the 211 patients who had received liver transplantation were applied for the established algorithms at the onset of hepatic encephalopathy, 141 patients (66.8%) were classified as category-1A, category-1B, category-1D, or category-1F, in which categories the mortality rates were greater than 50% in patients for the formation of the algorithm (Table 4a). In contrast, 53 patients (25.2%) were classified as category-1G, in which the mortality rates were 25.0 and 29.4%, respectively, in patients used for the formation and those used for the validation of the algorithm.

The outcome at 5 days after the onset of hepatic encephalopathy was assessed in 112 (53.1%) of the 211 patients who had received liver transplantation, because the transplantation was done within 5 days after the onset of hepatic encephalopathy in 99 patients (Table 4b). Consequently, 75 (67.0%) of the 112 patients were classified as category-2A, category-2B, category-2D, category-2E, or category-2H for the formation of the algorithm, in which categories the mortality rates were greater than 50%. Sixteen patients (14.3%) were classified as category-2C for validation of the algorithm, in which category the mortality rate was greater than 90%, despite the fact that the mortality in it was only 16.1% in the patients used for formation of the algorithm.

## Discussion

In the present study, we established a predictive model to determine the outcome of patients with ALF through decision tree analysis, one of the data-mining methods. Data-mining has been applied to analysis in fields such as business intelligence, marketing, banking and finance, customer relationship management, and engineering, as well as various areas of science, including medicine. In clinical medicine, data-mining techniques are used to construct a predictive model, which supports clinical decisions for researchers as well as practitioners [17]. A decision tree algorithm is one of the most popular data-mining techniques, constructed through recursive data partitioning, where the data are split according to the values of a selected attribute in iteration. Decision trees have already been applied to the field of hepatology; for example, to analyze the characteristic features of hepatocellular carcinoma [18–20], and to evaluate the therapeutic efficacy of pegylated-interferon and ribavirin for patients with chronic hepatitis due to HCV infection [21, 22].

In the present study, algorithms of two types were established; an algorithm for use at the onset of hepatic encephalopathy and one for use 5 days later, because, in Japan, conservative medical care including artificial liver support is generally performed in most patients, including those receiving liver transplantation, following the onset of hepatic encephalopathy. In fact, as shown in Table 1c, plasma exchange and hemodiafiltration were carried out in more than 90 and 70%, respectively, of patients with ALF. Thus, the outcome of the patients could be evaluated 5 days after the onset of hepatic encephalopathy in 53% of patients receiving liver transplantation (Table 4). The data sets obtained from ALF patients seen between 1998 and 2003 were used for the formation of the algorithms and those from the patients seen between 2004 and 2007 for their validation, because the outcomes of the patients seen in the two periods were almost equivalent, although there were some differences between the two periods in the frequencies of the therapeutic procedures undertaken (Table 1c, d).

According to the established decision tree algorithms, the patients with ALF were classified into 7 categories through 6 items at the onset of hepatic encephalopathy and into 8 categories through 7 items at 5 days after the onset of hepatic encephalopathy. Serum bilirubin concentration was selected as the first split item in the former algorithm, and the patients were further classified based on peripheral blood platelet counts, age, presence or absence of ascites, and the etiology of liver injuries. In contrast, the prothrombin time at 5 days after the onset of encephalopathy was the first split item in the latter algorithm, and the patients were then classified based on the serum bilirubin concentration and presence or absence of cerebral edema, liver atrophy, and cardiac and renal failure at the onset of encephalopathy or 5 days later. The interval between the onset of disease symptoms and hepatic encephalopathy has been considered to be one of the most important factors to determine the prognosis of ALF patients [4], and this factor was selected as a parameter in the previous guidelines [5]. The prothrombin time and the ratio of the direct-to-total bilirubin concentration at the onset of hepatic encephalopathy were previously selected as parameters as well [5]. However, these factors were not chosen as items responsible for the prognosis of ALF patients in our novel model established through decision tree analysis. These decisions are in line with findings in our previous report [7], in which ALF patients could be classified into three clusters independent of the interval between the onset of disease symptoms and the onset of hepatic encephalopathy, and the prognosis of the patients differed markedly among the clusters. Moreover, among 7 items in the algorithms at 5 days after the onset of hepatic encephalopathy, the extent of cerebral edema, renal failure, and heart failure may vary depending on the therapeutic devices used, especially regarding methods for artificial liver support (ALS) [23–25]. High-flow continuous hemodiafiltration (CHDF) and on-line hemodiafiltration (HDF) are much more effective than conventional HDF and CHDF [26, 27]. In the present study, most of the patients received conventional CHDF and HDF (data not shown), and such therapeutic devices were not selected as factors affecting the prognoses of the patients.

Certain characteristic features in both our algorithms are deserving of inclusion in the algorithms. First, the categories can be divided into 2 types depending on their mortality rates; the mortality rates in patients used for the formation of the model were greater than 66.7% in 4 categories in both algorithms, while they were less than 33.3% in the remaining 3 and 4 categories, respectively, in the algorithm used at the onset of hepatic encephalopathy and that used 5 days later. Secondly, 341 of the 371 patients used for the establishment of decision trees (91.9%) were classified into 4 major categories, in which the number of patients belonging to each category was greater than 30 in the algorithm at the onset of hepatic encephalopathy. Also, 325 patients (87.6%) were classified into 5 major categories in the algorithm at 5 days after the onset of hepatic encephalopathy. Considering these characteristic features of both algorithms, the novel model constructed through the decision tree analysis seems to be useful for the prediction of the outcome of patients with ALF, because the first characteristic above allowed the analysis to achieve high accuracy rates when the outcomes of the patients were predicted qualitatively as “death” or “survival”. In contrast, the second characteristic may enable us to obtain stable results for prediction even after the validation. In fact, as shown in Table 3, the predictive accuracies of both algorithms were high; 79.0 and 83.6%, respectively, in the algorithm at the onset of hepatic encephalopathy and that at 5 days later, when the outcome of patients belonging to the categories with mortality rates greater than 50% was predicted as “death”. Moreover, the sensitivity, specificity, PPV, and NPV values were greater than 75% in the algorithm at the onset of hepatic encephalopathy, and greater than 80% in the algorithm at 5 days later. Also, the mortality rates in patients used for the algorithm formation were similar to those in the patients used for the validation in each category, except for category-2C. As a result, the predictive accuracies were also high in patients used for the validation algorithm; 71.2 and 73.1%, respectively, in the algorithm at the onset of hepatic encephalopathy and that at 5 days later, when the outcome of patients was assessed qualitatively. Thus, it is concluded that the present model, consisting of 2 algorithms, may be useful to predict the outcome of ALF patients both quantitatively and qualitatively. Clinicians can obtain the predictive mortality rates of the patients depending on the categories to which the patients belong, and they can also predict the outcome as “death” or “survival” with satisfactory accuracies.

However, there are several weak points in both algorithms to predict the outcome of the ALF patient. Although the reproducibility of the algorithm at the onset of hepatic encephalopathy was generally good in each category, a 29.6% difference in mortality rates was found between the formation and validation data sets in category-1B. Also, there was a 75.6% difference between the two data sets in category-2C. Moreover, the validation could not be done in categories-2D and -2H, because no patients were classified in these categories in the validation groups, and a similar situation was found in the analysis of patients who had received liver transplantation. The significance of such minor terminal nodes (leaves) constructed with small numbers of patients should be further validated in patients enrolled in the nationwide survey since 2008.

Liver transplantation was performed in 221 (21.6%) of the 1,022 patients enrolled in the study. These patients were excluded from the subjects used for the formation and validation of the decision tree algorithms. However, we evaluated the possible outcomes of these patients using the established algorithms. To our surprise, as shown in Table 4, 33% of the transplanted patients were classified into the categories showing a predictive mortality rate of less than 50% both at the onset of hepatic encephalopathy and at 5 days later. We note particularly that there existed 53 of 211 transplanted patients (25.1%) belonging to category-1G, with predictive mortality rates of 25.0 and 29.0%, respectively, in patients used for the formation and those used for the validation of the algorithms. Thus, the clinical features of transplanted patients should, in the future, be evaluated retrospectively with reference to peripheral blood platelet counts and the etiology of liver injury, as well as serum bilirubin concentration, the items responsible for classification as category-1G. Also, it should be noted that 16 of 112 patients (14.3%) were classified as category-2C at 5 days after the onset of hepatic encephalopathy. The significance of category-2C, characterized by items such as cerebral edema, liver atrophy, and cardiac failure, should be investigated further.

In Europe and the United State, the indications for liver transplantation in patients with ALF have been evaluated based on the guidelines proposed by O’Grady et al. [28], in which the prognosis was estimated differently in patients with liver failure due to acetaminophen intoxication and those with liver failure caused by viral hepatitis and drug allergy-induced liver injury. In the former category of patients, the prognosis was estimated based on three parameters: arterial blood pH, peak prothrombin time, and the serum creatinine level. In contrast, in the latter category of patients, the prognosis was determined based on 5 parameters: etiology of the disease, age of the patient, the duration of jaundice before the onset of hepatic encephalopathy, peak prothrombin time, and the serum bilirubin level. Thus, the usefulness of our novel model based on the decision tree analysis should also be evaluated in ALF patients in Europe and the United States, especially in those with acute liver failure due to viral hepatitis and drug allergy-induced liver injury, in comparison with the guidelines proposed by O’Grady et al. [28]. However, it should be kept in mind that the purpose of our model is to predict the possible mortality rates of ALF patients, but not to determine the indication for liver transplantation automatically. In our model, cerebral edema and cardiac failure, which may disallow the patients from receiving liver transplantation, are included as split items. Liver transplantation cannot be performed for patients showing high mortality rates due to complications caused by ALF that correspond to items that are contra-indications for surgical procedures.

In conclusion, we have developed a novel model consisting of two algorithms for predicting the outcome of ALF patients at the onset of hepatic encephalopathy and at 5 days later, through decision tree analysis. This system may be useful to determine the indication for liver transplantation, because the mortality rates can be estimated by the algorithms with high accuracy rates, which were similarly high both before and after validation.

## Abbreviations

ALF: Acute liver failure
LOHF: Late-onset hepatic failure
HBV: Hepatitis B virus
SOM: Self-organizing map
DIC: Disseminated intravascular coagulation
PPV: Positive predictive value
NPV: Negative predictive value
HAV: Hepatitis A virus
HEV: Hepatitis E virus
ALS: Artificial liver support
CHDF: Continuous hemodiafiltration
HDF: Hemodiafiltration
